# Bibliometric trends of health economic evaluation in Sub-Saharan Africa

**DOI:** 10.1186/s12992-016-0188-2

**Published:** 2016-08-24

**Authors:** Karla Hernandez-Villafuerte, Ryan Li, Karen J. Hofman

**Affiliations:** 1 Office of Health Economics, London, UK; 2 Institute of Global Health Innovation, Imperial College London, London, UK; 3 Priority Cost Effective Lessons for System Strengthening, MRC / Wits Rural Public Health and Health Transitions Research Unit, Wits University School of Public Health, Johannesburg, South Africa

**Keywords:** Health technology assessment, HTA, Low- and middle-income countries, Resource allocation, Network analysis

## Abstract

**Background:**

Collaboration between Sub-Saharan African researchers is important for the generation and transfer of health technology assessment (HTA) evidence, in order to support priority-setting in health. The objective of this analysis was to evaluate collaboration patterns between countries.

**Methods:**

We conducted a rapid evidence assessment that included a random sample of health economic evaluations carried out in 20 countries (Angola, Botswana, Congo, Lesotho, Madagascar, Malawi, Mauritius, Mozambique, Namibia, Seychelles, South Africa, Swaziland, Tanzania, Zambia, Zimbabwe, Ghana, Kenya, Nigeria, Ethiopia, Uganda). We conducted bibliometric network analysis based on all first authors with a Sub-Saharan African academic affiliation and their co-authored publications (“network-articles”). Then we produced a connection map of collaboration patterns among Sub-Saharan African researchers, reflecting the number of network-articles and the country of affiliation of the main co-authors.

**Results:**

The sample of 119 economic evaluations mostly related to treatments of communicable diseases, in particular HIV/AIDS (42/119, 35.29 %) and malaria (26/119, 21.85 %). The 39 first authors from Sub-Saharan African institutions together co-authored 729 network-articles. The network analysis showed weak collaboration between health economic researchers in Sub-Saharan Africa, with researchers being more likely to collaborate with Europe and North America than with other African countries. South Africa stood out as producing the highest number of health economic evaluations and collaborations.

**Conclusions:**

The development and evaluation of HTA research networks in Sub-Saharan Africa should be supported, with South Africa central to any such efforts. Organizations and institutions from high income countries interested in supporting priority setting in Sub-Saharan Africa should include promoting collaboration as part of their agendas, in order to take advantage of the potential transferability of results and methods of the available health economic analyses in Africa and internationally.

**Electronic supplementary material:**

The online version of this article (doi:10.1186/s12992-016-0188-2) contains supplementary material, which is available to authorized users.

## Background

Allocation of scarce health care resources is a key challenge facing the health sector globally. Low and middle income countries (LMIC) in particular are experiencing a considerable increase in health expenditures per-capita [1]. For instance, in Thailand, Ghana and Ethiopia, health expenditures per-capita grew more than 195 % between 2003 and 2013. At the same time, many of these same countries are undertaking major reforms in pursuit of Universal Health Coverage [2]. As a result there is a need for health resource allocation strategies that are effective, efficient and equitable. As the historic analysis by Rebelo [3] shows, health economics has been fundamental in helping to break down the complexity of optimal health resource allocation, and health technology assessment (HTA) has been identified as one such tool to support priority setting processes [4, 5].

In the case of Sub-Saharan Africa, the number of studies that use HTA tools such as cost-effectiveness analysis to inform health priority setting has been increasing. According to the Centre for Reviews and Dissemination (CDR) database [6], more than 50 % of the articles published after 1994 relating to HTA analysis in Sub-Saharan Africa (literature reviews, economic evaluations and other applications of HTA tools) were published between 2008 and 2013. An analysis conducted by the Office of Health Economics (OHE) and NICE International that explored the demand for and supply of evidence-informed priority-setting in a selected group of LMIC suggests that human technical capacity remains a limiting factor to fulfilling the demand for health economic evidence to support the decision-making process [2, 7]. This capacity varies between countries in the Sub-Saharan African region, and progress is reflected for instance in the number of cost-effectiveness analyses registered in the NHS Economic Evaluation Database (NHS EED) whose authors are affiliated to South Africa [6]. This variation in capacity has also been documented in related fields such as biomedical and health research publications [8, 9]. This is reflected in the analysis by Wagstaff and Culyer [10], which pointed out large differences among African countries in terms of the number of health economic articles produced. Such variations suggest that Sub-Saharan Africa countries with less capacity might benefit from adopting, adapting and contextualising transferrable evidence generated by neighbouring countries in the region, and be more able to set their own evidence-informed priorities in health despite being less capable of producing their own economic and clinical evaluations of health interventions.

One question is whether the evidence collected and the decisions adopted in one context are applicable to another. In this regard, the Sub-Saharan African countries share similarities not only in terms of epidemiological profiles, but also in terms of the macro-level decision making processes and related macro-level contextual factors that affect decision making. Macro-level decision-making processes are defined as those concerning the organisation and the architecture of a health care system; for example, those related to service delivery arrangements, the organisation of the health workforce, health information systems and disease priority setting [11]. A qualitative study conducted by Jenniskens, Tiendrebeogo [12], in which stakeholders from five African countries were interviewed, reflects their similarities in terms of contextual factors, health priorities, accessibility to services and quality of health care. Given the similarities in epidemiological profile and contextual factors, it is expected that the countries with less capacity would benefit from using the available evidence as well as the decisions made in other Sub-Saharan African countries.

Replicating HTA that has already been conducted elsewhere would be worthwhile only if the expected value of improved decision making (based on more accurate, contextualized evidence) outweighed the costs of repeating the exercise specifically for local conditions. Instead, coordinating efforts to collect and share knowledge could enhance the local decision making process, allowing the results of HTA evidence to be adopted or adapted in a way that more accurately reflects local contexts without a prohibitive increase in costs. In this regard, the similarities as well as differences in capacity between the Sub-Saharan African countries open the door to possible mutually supporting streams of HTA-based activities with potential for cross-transferability. Even more important, the capacity gaps linked to the generation and interpretation of economic and clinical evidence for HTA could be partially overcome by the transfer of information between those African countries with social and economic similarities.

In addition, the findings of Kularatna, Whitty [13] suggest that health state valuations (e.g. EQ-5D and health state preference studies) from LMICs are scarce; of the 17 health state valuations in LMICs only two were from Africa [13]. Furthermore, the quality of the evidence is also a concern, and a systematic review of economic evaluations in Ghana suggested that a more coordinated African approach to the assessment of new health treatments could potentially improve the quality of the evidence for the region [14]. A successful example of a coordinated regional approach is HTAsialink. This is a collaborative networking organization consisting of 15 institutions that includes Asia’s HTA agencies, representatives of Asian Ministries of Health and two organizations outside Asia: the Australian Safety and Efficacy Register of New Interventional Procedures - Surgical (ASERNIP-S) and the UK’s National Insitute for Health and Care Excellence (NICE). Currently, HTAsialink members are collaborating in a research project to define the social value of the QALY through the analysis of household surveys across Asia [15]. Furthermore, HTAsialink produces an electronic newsletter which is distributed to the members to share information and strength the collaboration between the members [16].

In this context, it would be useful to examine collaborations between Sub-Saharan African health economic researchers. There is currently little evidence of the specific effects of collaboration on the transferability of HTA evidence and on the improvement of the countries’ capacities to develop HTA programs. Nevertheless, the effect of collaboration on research outputs has been investigated in different areas of health research. For instance, Lachat, Roberfroid [17] examined the network of African researchers collaborating on the topic of nutrition, and the results suggest that this has not achieved its full potential. Similarly, collaboration patterns of the cardiovascular disease research in Africa suggest that limited resources to support research in Africa could be enhanced by leveraging closer partnership between African researchers [18]. The literature also suggests that collaboration improves transferability of information in different fields of research. This has been shown for genetics, drug development and nanotechnology [19–23]. Collaborations between researchers trigger spillovers in the form of expansion of ideas and advancement of research, and it would be reasonable to assume that the same could apply to HTA research.

The study of co-authored publications is a standard way of measuring research collaborations [17, 24–26]. The present analysis explores the relationships between Sub-Saharan African health economic researchers and their respective co-authors. Collaboration patterns between countries might indicate the extent to which the generation and transfer of evidence could support decision making processes in health priority setting.

Little is currently known about the patterns of collaborations in HTA or economic evaluations of health care interventions. This is the first study that explores the geographic network of the Sub Saharan African researchers in relation to HTA.

There were two main objectives of the study. First, to consider the potential in the region to take advantage of the results and methods of available HTA analysis. In order to do so, we conducted a rapid evidence assessment of a representative sample of academic articles that applied HTA (specifically economic evaluations) in Sub-Saharan African countries. Second, to analyse the main trends of the HTA literature about Sub-Saharan Africa in terms of disease focus, types of intervention evaluated, journal impact factors, and patterns of collaboration between authors within and outside of Sub-Saharan Africa. These will provide an indication of the level of advancement in health economic evaluation, the topic areas in which the evidence is growing, and elucidate the potential for transfer of evidence.

## Method

### Rapid evidence assessment

Rapid evidence assessments and systematic reviews are both systematic processes of gathering and reviewing evidence [27, 28]. The difference between the two lies in that rapid evidence assessment is time-constrained and usually aimed at capturing the key issues reflected in the evidence, while a systematic literature review should exhaust all the possible sources of information with less consideration of the time and resources spent, and indeed whether any additional information retrieved provides sufficient value to outweigh the additional costs required in evidence search and analysis.

There are limitations in rapid evidence assessment. First, this method does not cover all available literature, and could result in important evidence being missed from the analysis. Similarly, rapid evidence assessment does not include grey literature. This means that economic evaluations available for the decision makers but not published in peer-reviewed journals are not reflected in the conclusions. After considering the relative costs and benefits of both methods, we selected rapid evidence assessment as the more efficient and useful method for the current analysis.

### Selection of articles

An electronic search for articles was conducted using the NHS EED [6]. This comprehensive database includes over 16,000 economic evaluations of health care interventions worldwide. Through an ongoing search of articles in MEDLINE, EMBASE, CINAHL, PsycINFO and PubMed, NHS EED has collected all articles in which the costs and outcomes of two or more interventions are compared using cost-benefit, cost-utility, or cost-effectiveness analyses. Given that the articles collected in the NHS EED match our objectives, we based the rapid evidence assessment on NHS EED. Note that the NHS EED does not include systematic literature reviews.

In order to identify economic evaluations conducted on Sub-Saharan Africa, our search terms were the names of the 15 countries that are members of the Sub-Saharan African Development Community (SADC): Angola, Botswana, Democratic Republic of the Congo, Lesotho, Madagascar, Malawi, Mauritius, Mozambique, Namibia, Seychelles, South Africa, Swaziland, Tanzania, Zambia and Zimbabwe. In addition, given HTA has seen considerable development in Ghana, Kenya, Nigeria, Ethiopia and Uganda these five countries were also included. Since NHS EED includes only economic evaluations, our search criteria did not include any additional words, such as those related to priority setting, HTA or economic evaluation.

The search was conducted with no language restriction, but limited to documents included in the NHS EED database before February 2015 and published between January 2004 and December 2014.

In order to obtain a representative sample with a manageable number of economic evaluations for every country, we followed a three step selection process:

Step 1. The names of the selected countries were searched one-by-one using the option “Any field” (i.e. titles, abstracts and full texts).

Step 2. A subsample of articles was extracted separately for each of the countries, such that a maximum of 10 articles per country was selected. Based on the number of hits in Step 1, one of the following two actions was carried out:

- For those countries in which the number of hits was smaller than or equal to 10, all hits were included in the subsample and the duplicated articles were excluded.
  Table 1 shows the results for the 9 countries in which no more than 10 articles were found during the search. No economic evaluations were identified for Angola and Mauritius, thus these two countries are not considered in the remaining analysis.

**Table 1:** Selection of articles

Country	(a) Articles listed in the NHS EED database (# of hits)	(b) Name of the country in the title	(b) Duplicated articles	(b) Number of selected articles	(c) Not international Affiliation	(c) Only mentioned in passing	Articles Included in the analysis
Sub-Saharan African countries with fewer than 10 articles listed in the NHS EED database							
Angola	0			0			0
Botswana	5			5			5
Congo	5			5	2	1	2
Lesotho	3		1	2	1		1
Madagascar	2			2			2
Mauritius	0			0			0
Namibia	2		1	1	1		0
Seychelles	2			2			2
Swaziland	3		1	2	1		1
Sub-Saharan African countries with more than 10 articles listed in the NHS EED database							
Malawi	11	7		10			10
Mozambique	13	8		10			10
South Africa	105	53		10			10
Tanzania	34	20		10			10
Zambia	26	15		10			10
Zimbabwe	13	5	3	10	1	1	8
Ghana	13	11		10			10
Kenya	40	20		10			10
Nigeria	23	16	2	10			10
Ethiopia	11	8		10	1	1	8
Uganda	56	35		10			10
Total	367						119
- For those countries with more than 10 hits, the search was narrowed to the articles in which the name of the country was mentioned in the title.
  I. If the number of articles in which the name of the country was mentioned in the title was greater than 10 (South Africa, Tanzania, Zambia, Ghana, Kenya, Nigeria and Uganda, see Table 1 column “(b) Name of the country in the title”), 10 articles were randomly selected, and each assigned a number using the function “sample()”of the program R 3.2.3 (The R Core Team, 2016).
  II. If the number of articles in which the name of the country was mentioned in the title was 10 or fewer (Malawi, Mozambique, Zimbabwe and Ethiopia, see Table 1 column “(b) Name of the country in the title”) all the articles were selected.
  III. Duplicated articles were excluded (see Table 1 column “(b) Duplicated articles”).
  IV. With the objective of reaching 10 articles per country, articles were randomly selected among those not previously selected in I and/or II (meaning those articles in which the name of the country was not included in the title) (see Table 1 column “(b) Number of selected articles”).
  Table 1 also shows the results for the 9 countries in which more than 10 articles were found during the search.

Step 3. After subsamples of articles were selected for all countries, we excluded those articles where:

- none of the first three authors’ institutional affiliation was in Sub-Saharan Africa, or
- the analysis was not focused on countries in Sub-Saharan Africa, or
- the country was mentioned only in passing in the full text, meaning that the economic evaluation pertained to a different country.

**Fig. 1:**
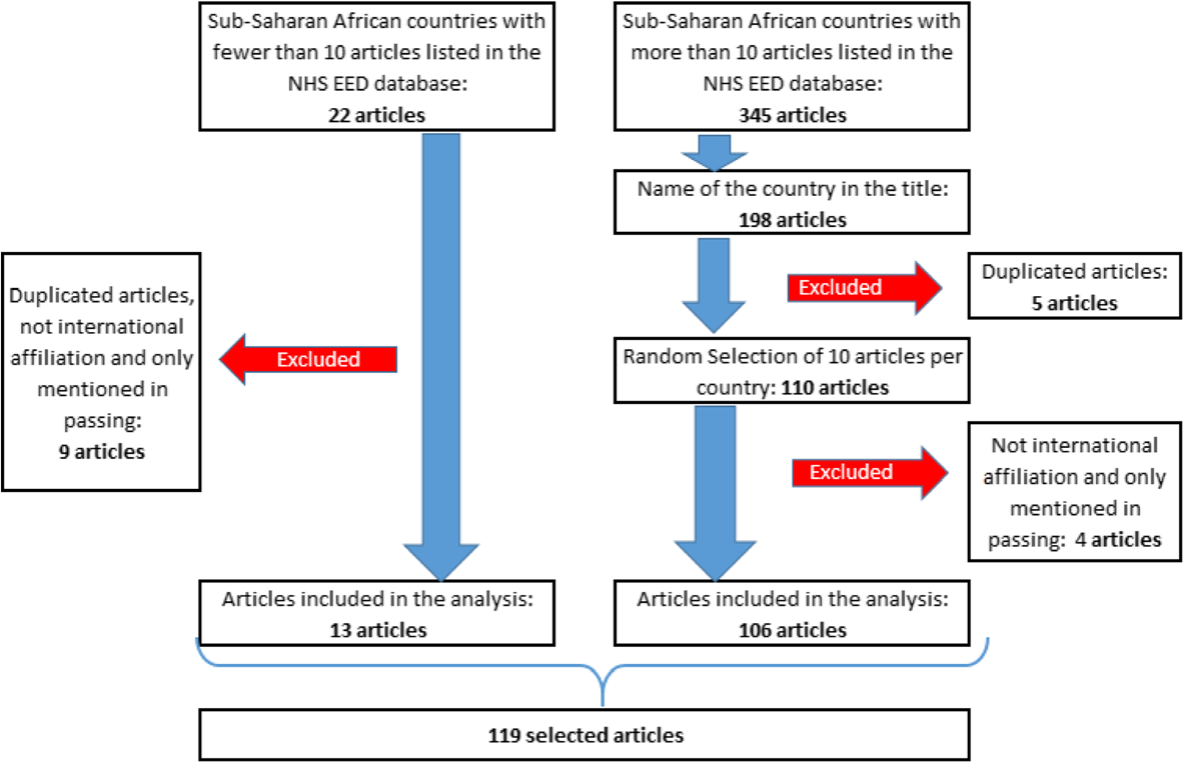
Flow of studies in rapid evidence assessment

Table 1 shows for each of the three steps of the selection process the number of selected articles per country. 119 articles were selected from which 13 articles pertained to more than one Sub-Saharan African country. A full list of the articles that include an author from more than one country can be found in Appendix, Table 5. In the case of Namibia and Swaziland, all the included articles consider more than one Sub-Saharan African country. Figure 1 presents the steps followed during the selection of the 119 articles.

For each of the 119 articles four categories of information are extracted: 1) general information (i.e. title, publication year, method, and journal of publication); 2) geographic scope; 3) disease and type of intervention; and 4) authors’ countries of affiliation. A detailed description of the information collected in each category is presented in Appendix, Table 6.

### Country of affiliation and network of the Sub-Saharan African researchers

Next we explored the network of researchers with a Sub-Saharan African. The research network was considered an indicator of the cooperation between research workforces of the countries. Approximately one third of articles (42/119) have a first author with an affiliation from at least one Sub-Saharan African country (Appendix, Table 7). Three of 42 appear twice as first authors, thus the final subsample is equal to 39 different authors.

Co-authorship on scientific publications, a proxy for collaboration [17], was used as the basis of the research network analysis. We searched PubMed for all articles published by all 39 authors between 1990 and 2014, using the full name of the author (as appearing in the selected article) as the search term. The 729 identified publications are analysed hereafter as ‘network-articles’.

The first author of each network-article is considered the main researcher of that study, and therefore also considered the strongest collaborator. The name and country of affiliation of the first author of each network-article was extracted. In those cases in which the first author of the network-article was the same as the author of the NHS EED subsample, the country and name of affiliation of the second author was used.

In order to examine for each first author the concentration of collaborations per country, we estimated the Herfindahl Index ($HI$). The Herfindahl Index is interpreted here as the concentration of co-authors within any given country, ranging 0 (having co-authors from a large number of countries) to 1.0 (having co-authors only in the same country). It is estimated as $HI=\sum_{i=1}^N{s}_i^2$ where ${s}_i$ is the share of articles in which the co-authors’ affiliation country is $i$ as part of the total number of articles (N) published by the author and registered in PubMed.

In order to illustrate collaboration patterns and the potential for information transfer between countries, we plotted a connection map reflecting the number of paper published by the 39 first authors, their countries of affiliation, and connections with researchers inside and outside their countries.

## Results

### Disease focus of studies

Among the 119 sampled economic evaluations of health interventions, most were related to four communicable diseases: HIV/AIDS (42/119, 35.29 %), malaria (26/119, 21.85 %), tuberculosis (6/119, 5.04 %) and diarrhoea (4/119, 3.36 %).

A small percentage (7/119, 6 %) of the articles were not related to any particular disease. These can be classified into three groups, first those articles relating to the health system organisation. For instance, the analysis conducted by Curry, Byam [29] studied the impact of large-scale interventions on primary care services for women and children in low-income and rural areas. The second group evaluated preventative interventions, but without a clear link to any particular disease, such as the work done by Hu, Grossman [30] in which three safe abortion methods were compared with unsafe abortion in terms of cost-effectiveness. A third group examined methodological approaches. For instance, the investigation conducted Hansen and Chapman [31] who studied the feasibility of conducting cost-effectiveness analyses for a large number of health interventions in a developing country using a consistent methodology.

Regarding conditions other than communicable diseases, the sample included 3.36 % (4/119) of studies on cancer (breast and cervical cancers), 2.52 % (3/119) on acute malnutrition and 1.68 % (2/119) on maternal mortality. There was one example for the prevention of cardiovascular disease, and another for the use of antihypertensive medications. The former analysed the cost-effectiveness of singlerisk-factor management in comparison to management based on total cardiovascular risk factors in Seychelles [32]. The latter evaluated the cost-effectiveness of 4 classes of medications commonly used in Nigeria [33].

### Types of intervention studied

**Fig. 2:**
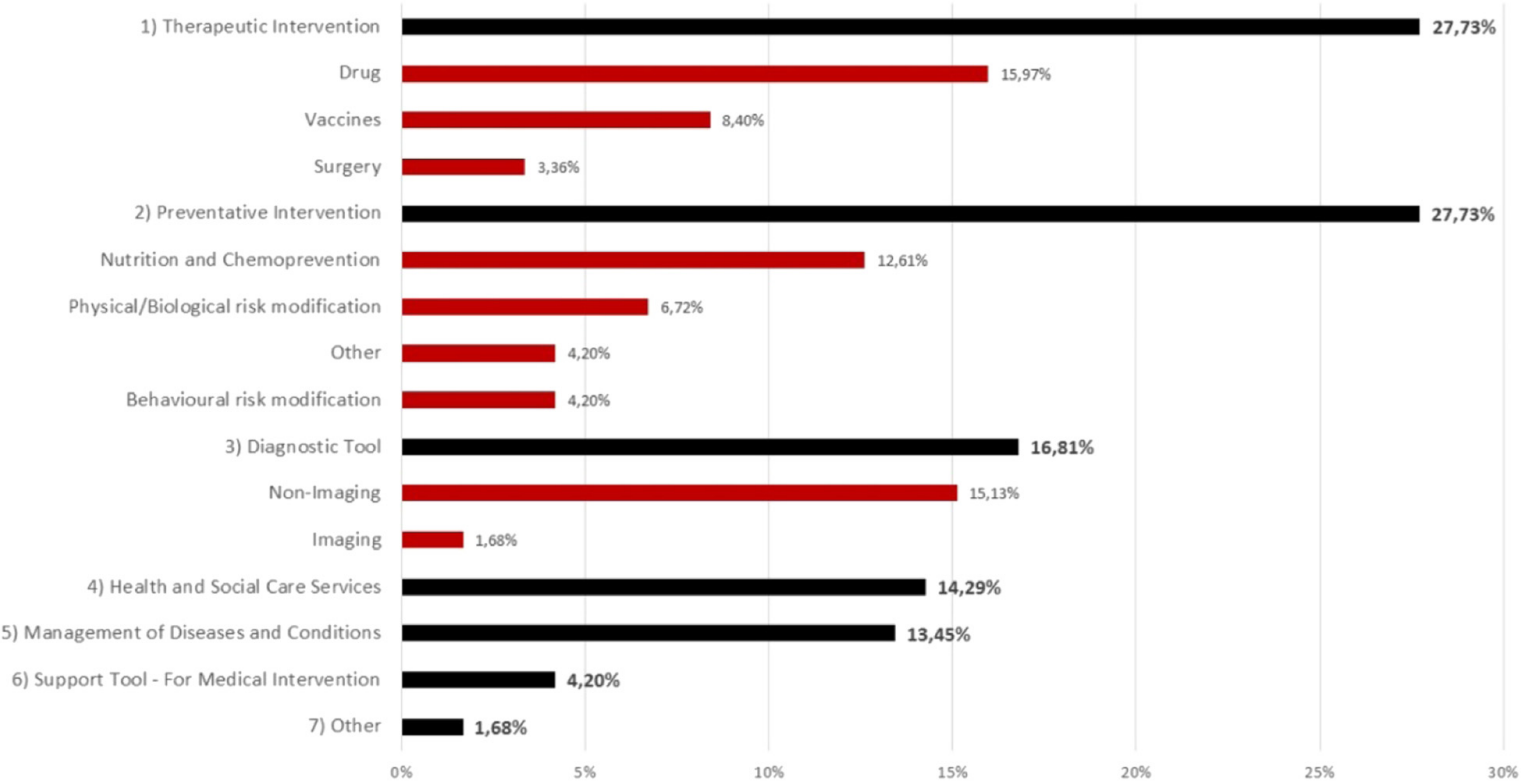
Number of included articles by type of intervention. Source: Authors’ Elaboration

Figure 2 classifies the sample of articles according to the criteria used by the UK Medical Research Council (MRC) [2]. This classification system breaks down the health interventions into seven categories and 19 subcategories (Appendix, Table 7). Figure 2 shows that the two most common type of interventions analysed were therapeutic interventions and preventive interventions. Out of the 27.73 % (33/119 articles) of observations classified as therapeutic interventions, 15.97 % (19/119) were economic evaluations of the introduction of a new drug. Among preventive interventions, nutrition and chemoprevention comprised the highest proportion of observations (16/119). Chemoprevention refers to the use of pharmacologic or natural agents for the purpose of preventing disease or infection, and 11/16 articles analysed the effect of HIV chemoprevention [34–43]. Moreover, more than half the articles in the subcategories “nutrition and chemoprevention” and “introduction of a new drug” corresponded to treatments to prevent HIV transmission. Finally there was a sizeable proportion of economic evaluations (18/119, 15.13 %) analysing non-imaging diagnostic tools.

### Quality of publications

Journal impact factor is commonly considered as a proxy for publication quality. The 119 selected articles were published in 51 different journals; 8 of these journals did not have a reported impact factor from International Scientific Institute (ISI) [44]. 60 % of the selected articles were published in 12 journals (see Table 2). *PLOS ONE* (15.1 %), *Cost Effectiveness and Resource Allocation* (9.2 %) and *Malaria* (8.4 %) comprised the highest share of articles. The impact factors of the two first journals did not exceed the weighted average impact factor of the sample. The Cost Effectiveness and Resource Allocation journal does not have a reported impact factor; however, the value of the unofficial impact factor is 1.16 [45] which is also below the average level.

**Table 2:** Authors by Region of Affiliation (# Authors)

	First author	Second author	Third author
Africa	31	50	54
Outside Africa	77	62	54
More than one affiliation and at least one from Africa	11	6	4
Total	119	118	112

### Network analysis

Although all the 119 articles were about Sub-Saharan Africa, the majority of the first authors (77/119, 64.7 %) were affiliated to an institution outside Africa (Table 2). This proportion was slightly smaller but still sizeable among the second authors (62/118 authors, 52.5 %) and third authors (54/112, 48.2 %).

Based on authorship, two countries are the leaders in economic evaluations in Sub-Saharan Africa: the USA and the UK. 43.3 % of the first three authors and 52.1 % of the first authors were from one of these two countries (Appendix, Table 7).

For the network analysis, we selected the 39 first authors with an affiliation from Sub-Saharan Africa, comprising 729 network-articles in total. The average of network-articles per author was 18.69 (range 1 and 81) (Table 3). Out of the 20 countries included in the original sample, 14 were represented by at least one of the 39 first authors (Botswana, Congo, Ethiopia, Ghana, Kenya, Lesotho, Madagascar, Malawi, Mozambique, Nigeria, South Africa, Tanzania, Uganda, and Zambia).

**Table 3:** Number of network-articles and country of affiliation of the authors selected for the network analysis

Author	Affiliation Country	# of Network-Articles	Co-authors’ affiliation Country	Herfindahl Index	Co-authors’ second affiliation country*
Akumu, Angela Oloo	Kenya	2	Kenya	1.00	
Bikilla, Asfaw Demissie	Ethiopia/Norway	2	Norway	1.00	
Ekwunife, Obinna Ikechukwu	Nigeria	14	Nigeria	1.00	
Kifle, Yibeltal A	Ethiopia	1	Ethiopia	1.00	
Kivuti-Bitok, Lucy W	Kenya	3	Kenya	1.00	
Ogu, Rosemary	Nigeria	8	Nigeria	1.00	
Opondo, Everisto	Kenya	2	Kenya	1.00	
Pemba, Dylo F	Malawi	2	Malawi	1.00	
Suleiman, Ismail Ayinla	Nigeria	2	Nigeria	1.00	
Tekeste, Asayehegn	Ethiopia	1	Ethiopia	1.00	
Leisegang, Rory	South Africa	7	South Africa/USA	0.72	
Uzochukwu, Benjamin	Nigeria	66	Nigeria/UK	0.69	
Ezenduka, Charles	Nigeria	5	Netherlands/Nigeria	0.68	
Mori, Amani T	Tanzania/Norway	6	Norway/Tanzania	0.63	Norway
Samandari, Taraz	Botswana/USA	26	Botswana /Botswana /South Africa /UK /USA	0.61	USA
Lemma, Hailemariam	Ethiopia	7	Ethiopia /Sweden	0.56	
Drain, Paul K.	Madagascar	27	Canada/Madagascar/South Africa/Swaziland/USA	0.55	Cuba
Tumwesigye, Nazarius M	Uganda	36	Australia/Ireland/Switzerland/UK/Uganda/USA	0.54	Sweden
Nonvignon, Justice	Ghana	16	Congo/Ghana/Nigeria/USA	0.52	
Badri, Motasim	South Africa	53	Saudi Arabia/South Africa/UK/USA	0.52	Japan /UK
Schnippel, Kathryn	South Africa	11	South Africa/USA	0.51	USA
Matangila, Junior R	Congo/Belgium	2	Belgium/Congo	0.50	
Onwujekwe, Obinna	Nigeria	81	Nigeria/South Africa/Sudan/UK /USA	0.47	
Zikusooka, Charlotte M	South Africa	6	South Africa/Uganda/USA	0.44	
Zurovac, Dejan	Kenya	32	France/Kenya/UK/Uganda/USA/Zambia	0.44	UK
Osei-Kwakye, Kingsley	Ghana	13	Gabon/Germany/Ghana/UK	0.44	
Díez-Padrisa, Núria	Mozambique/Spain	11	Mozambique/Spain/USA	0.39	Spain
Duffy, Kevin	Uganda	11	Germany/UK/Uganda/USA	0.39	
Ansah, Evelyn K.	Ghana/UK	15	Ghana/Malawi/Netherlands/UK	0.38	
Rosen, Sydney	South Africa/USA	57	France/South Africa/USA/Zambia	0.38	USA
Nthumba, PM	Kenya	19	Kenya/Spain/Switzerland/USA	0.38	
Olusanya, Bolajoko O.	Nigeria/UK	49	Benin/Germany/Nigeria/South Africa/UK/USA	0.36	
Mbonye, Anthony K.	Uganda	30	Canada/Denmark/UK/Uganda/USA	0.34	South Africa
Chanda, Pascalina	Zambia	11	Kenya/South Africa/UK/USA/Zambia	0.28	
Chhagana, Meera K	South Africa	10	Norway/South Africa/Switzerland/USA	0.28	
Albert, Heidi	South Africa	12	Pakistan/South Africa/Switzerland/Uganda/USA	0.26	
Armstrong Schellenberg, Joanna RM	Tanzania/UK/Switzerland	39	Brazil/Gambia/Spain/Switzerland/Switzerland/Tanzania/UK/USA	0.22	Netherlands/Spain/UK
Jouquet, Guillaume	Lesotho	8	Belgium/Lesotho/South Africa/Swaziland/Switzerland	0.20	
Kolaczinski, Jan H	Uganda/UK	26	Australia/Ethiopia/France/Kenya/Pakistan/South Sudan/UK/Uganda	0.19	Switzerland/UK/USA
	Total	729	Average Herfindahl Index	0.59	

*The second country of affiliation of a co-author is not considered in the estimation of the Herfindahl Index or in the network map

The majority of the collaborations were with a researcher from Europe (principally the UK), the USA or within Africa. Regarding the connections inside Africa, network-articles included those published with co-authors from Nigeria (176/ 729), South Africa (95/729) and Uganda (68/729). There were only a few connections with Australia and Asia (Pakistan and Saudi Arabia).

Table 3 shows the Herfindahl Index for each author. Note that the authors with the highest number of articles did not have the lowest Herfindahl Indices, implying that a well-established academic career does not necessarily correspond with having research collabrators with a large number of countries.

**Fig. 3:**
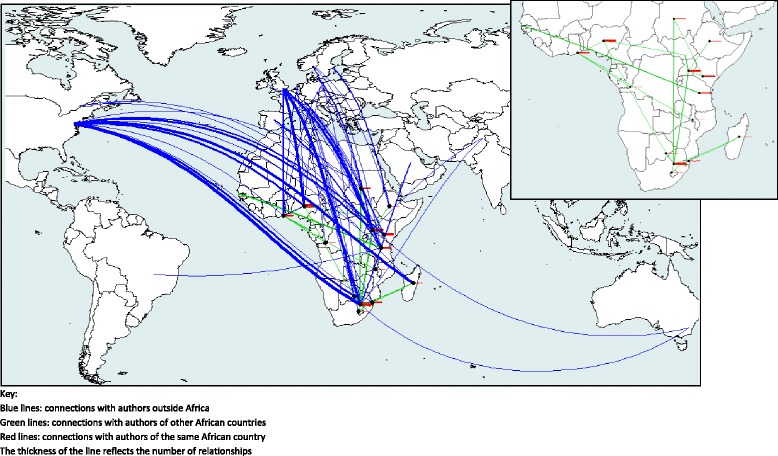
First author relationships according to affiliation country

Figure 3 presents the connection map reflecting the number of papers published and the countries of affiliation. The black points mark the countries to which the 39 first authors are affiliated (Table 3). Note that the connections are analysed at country level but visually represented on the map as connections between capital cities.

The thickness of the red lines clearly shows that the Sub-Saharan African authors are collaborating first with authors of their own country. More than 65 % of the network-articles with an author affiliated to an institution in Botswana, Kenya, Madagascar or Nigeria had the first two authors affiliated to institutions in the same country. For Uganda, the first two authors were affiliated to institutions in the same country in 50 % of the network-articles.

There was also a strong connection between Sub-Saharan African researchers and researchers from the USA and Europe, particularly the UK, which is observed in the blue lines. Regarding the USA, the highest number of links was found with South Africa (30/124) and Madagascar (16/22). In the case of the UK, the higher number the network-articles was observed with South Africa (24/124) and Nigeria (14/102). We also found a sizeable proportion of the network-articles of Ghana and Tanzania linked to the UK.

Additionally, the number and thickness of the green lines indicates weak collaboration between Sub-Saharan African researchers of different countries. Even among authors of South Africa and Uganda, who were highly active in the production of articles, there was little indication of collaborations with other African countries. However, the strongest connection between two Sub-Saharan African countries occurred between South Africa and Uganda, with seven network-articles.

**Fig. 4:**
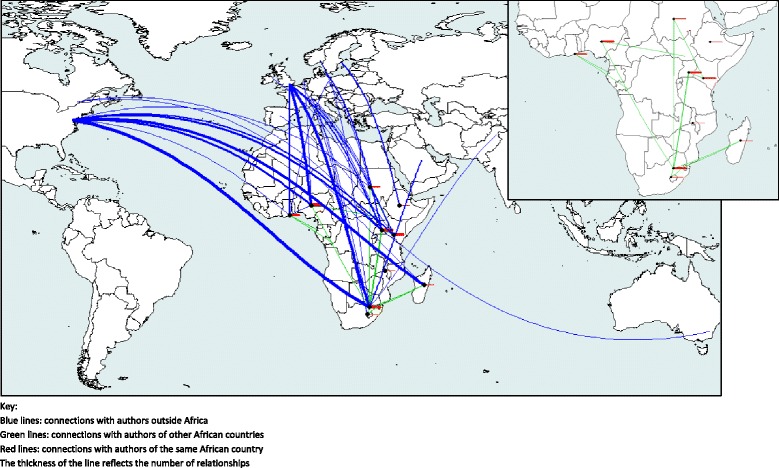
First author relationships according to affiliation country and excluding researchers with more than one affiliation

Out of the 39 authors, 10 had two affiliations, of which one was from a country outside Africa (USA, Belgium, Norway, Spain or the UK). This could distort the findings since such authors would be expected to have more co-authors from outside Africa. In order to address this bias, Fig. 4 shows the connection map excluding the network-articles related to these 10 authors. An important number of the connections between the USA and South Africa, and between Uganda and the UK, disappeared. Most important, the number of connections between the African countries also decreased, such as those with Ethiopia. Nevertheless, the pattern is the same as in Fig. 3: the strength of collaborations was clearly weaker between the African countries in comparison with the collaborations outside Africa.

## Discussion

To our knowledge, this is the first published study aimed at identifying patterns of collaboration between health economic researchers in Sub-Saharan Africa, and also the first study to apply bibliometric network analysis techniques within the health economic evaluation literature. Our analysis suggests that collaboration between researchers among Sub-Saharan African countries is weaker than that between the Sub-Saharan African researchers and their counterparts in USA and Europe, particularly UK. This is similar to the results of Chuang, Chuang [8] who applied a bibliometric analysis to public health research in Africa, and found that the main collaborators of East and South Africa were researchers from the USA and the UK, while there was almost no connection with countries in Asia. Similarly, Beattie, Renshaw [46] showed that the highest number of collaborations between African countries and countries outside the region related to research in malaria were with UK and USA. Our findings are consistent in suggesting that the assessment of health technologies through the application of economic evaluations in Africa appear to be influenced particularly by two countries, USA and UK.

Our rapid evidence assessment suggests that among Sub-Saharan African countries, South Africa has the highest production of health economic evaluations as well as the highest number of connections with other African countries. This is in agreement with the analysis by Wagstaff and Culyer [10] which found South Africa to be the most productive among 25 countries in terms of number of publications and citations related to health economics. We found health economic evaluations relating to all Sub-Saharan African countries with the exception of Angola and Mauritius, however the quantity varied considerably among the countries, from only two registered studies (Madagascar, Namibia and Seychelles) to more than 100 (South Africa). The apparent special status of South Africa is consistent with that identified in other studies of health research networks in Africa. Ettarh [18] found that the strongest connections on cardiovascular research occurred between South Africa and Nigeria; and Onyancha [47] who analysed HIV/AIDS collaboration networks in Sub-Saharan Africa and concluded that while Uganda presented the largest number of author networks, it was also the main research focus of the South African research network, the other major HIV/AIDS research network in the region.

Taken together, our findings suggest substantial variation in HTA capacity among Sub-Saharan African countries, that South Africa’s health system is better developed to support evidence-based decisions in comparison with the other countries, and South Africa would be well-placed to consider as a centre for the creation of a regional HTA network in Sub-Saharan Africa. Such a network could facilitate the strengthening of capacity in countries with fewer resources and less experience in the production and use of health economic evaluations. The kinds of capacity that might be lacking include data capacity, technical capacity of researchers to conduct economic evaluations, capacity of policymakers to commission, interpret and use evidence, and the capacity of the health system to incentivise relevant research and knowledge translation [48]. These various capacity gaps need to be addressed accordingly in order to foster the production of relevant and high quality economic evaluation that can support the policy makers’ decisions in health priority-setting.

Our results show that most of the economic evaluations conducted in Sub-Saharan Africa is biased towards treatments of communicable diseases, in particular HIV and malaria. This reflects the health priorities of the Sub-Saharan African countries since communicable diseases are among the five leading causes of disease burden as well as having significant humanitarian and economic impact in Africa [49]. Sub-Saharan Africa has the highest prevalences of HIV worldwide [50], with more than 19 million people living with HIV in the countries listed in Table 4 (and more than 10 % of the population in countries such as Botswana, Swaziland, Lesotho and South Africa) [50]. Regarding malaria, the WHO estimates that Africa accounted for 188 million cases of malaria and 292,000 deaths in children aged under 5 years in 2015 [51]. Moreover, since HIV and malaria were central to the Millennium Development Goals (MDGs), national and international funders have devoted a large proportion of aid investments to preventing and treating such diseases especially in Africa [52, 53]. For instance, in Kenya, Ghana, Uganda, Nigeria, Mozambique and Malawi less than 15 % of the health expenditures related to HIV were from domestic public funds [50]; and despite dramatic decreases in malaria prevalence in countries such as Botswana, Namibia, Ethiopia, Zambia, Zimbabwe, Swaziland and South Africa over the past 15 years, healthcare expenditures destined to malaria treatment and control still represents substantial proportions of national health expenditures, which are mostly covered by international funds [51].

**Table 4:** Journals and impact factors related to the articles in the sample

Journal	Articles		Impact Factor*
	Number	% of the sample	
PLOS ONE	18	15.1 %	3.54
Cost Effectiveness and Resource Allocation	11	9.2 %	NA
Malaria Journal	10	8.4 %	3.49
Journal of Acquired Immune Deficiency Syndromes	9	7.6 %	4.39
PLOS Medicine	4	3.4 %	14.0
Health Policy and Planning	4	3.4 %	3.00
Tropical Medicine and International Health	4	3.4 %	2.30
AIDS	3	2.5 %	6.56
Bulletin of the World Health Organization	3	2.5 %	5.11
Value in Health	3	2.5 %	2.89
American Journal of Tropical Medicine and Hygiene	3	2.5 %	2.74
Weighted average impact factor for the full sample (weighted by the number of articles)			4.959
Maximum impact factor for the full sample			39.207 (Lancet)
Minimum impact factor for the full sample			0.191 (Malawi Medical Journal)

*SCI Journal Impact Factor: Measure that reflects the average number of citations [44]

Nonetheless, there were some studies of non-communicable diseases in our sample and these may increase in importance as the health priorities of Sub-Saharan African countries are changing [33]. Additionally, the results also indicate that the assessment of drugs and non-imaging diagnostic tools are the two most common types of technologies analysed.

This study had several limitations. First, from the initial sample of articles, only the first authors were considered in the network analysis. Most of the economic publications recognize the participation of at least four authors. In some of the studies included, the Sub-Saharan African authors do not appear in the first three names in the list of co-authors. To consider the research network of all African co-authors could allow a more robust examination of the patterns potentially useful for transferring knowledge. This is because these authors have also acquired experience and skills that could be useful for the development of HTA in Sub-Saharan Africa. Moreover, in some publications the first author is not the main researcher since some economics journals list author names in alphabetical order. Second, the analysis did not explored the transferability of the evidence itself, the extent to which the methods applied in one African country can be applied to other country, or the demand side of HTA evidence (policy decision makers). Contextual factors could hinder transferability of and positive spillover effects of generated research, and without a strong political commitment production of HTA evidence in unlikely to have a positive impact on the effectiveness and efficiency of health priority-setting [54]. Third, the use rapid evidence assessment meant that not all the available literature was covered and this could have resulted in a bias in the findings. However, this limitation was partially compensated by the fact that NHS EED comprises an exhaustive search of articles in MEDLINE, EMBASE, CINAHL, PsycINFO and PubMed. The NHS EED database includes over 16,000 economic evaluations of health care interventions worldwide reduced the likelihood of missing key articles that could have an effect on the results. Furthermore, we used random sampling to ensure that the included articles were representative of publications in Sub-Saharan Africa. Nevertheless, potentially relevant grey literature such as country reports and book chapters was not considered in this analysis; we recommend for future investigation a systematic review of the grey literature that, giving the lack of publicly available evidence, considers measures to overcome the difficulties in the collating economic evaluations in Africa. Fourth, as a proxy for publication quality we used the International Scientific Institute impact factor, but this did not include all the journals in which the selected articles are published. Therefore, future studies should consider also other appropriate measures of publication quality.

## Conclusions

The need for setting priorities in health within limited resources has stimulated the search for evidence-based tools, such as HTA, to inform health policymakers during the decision making process. Despite the increasing attention of the scientific community on HTA, in Africa the production of HTA analysis has been characterized by lack of resources and human capital. Given that collaboration between researchers has been an important element in the diffusion of knowledge in several fields of study, it could be also a key factor to overcoming the problems that generation and transferability of evidence to support health priority setting in Africa.

Our study highlights important challenges facing the transfer of HTA information between Sub-Saharan African countries, as well as important opportunities. Collaboration between Sub-Saharan African researchers is important in the generation and transfer of evidence to support the decision making process for health priority setting. However, collaboration between African institutions is currently weak and research appears to be heavily dependent on collaboration with Europe and North America. There are various possible explanations, of which we consider two as the most plausible. First, the large amounts of international funds invested in improving health in Sub-Saharan Africa increases the interest of researchers in development countries to conduct projects with a focus on the region. Second, those African researchers that have studied in American or European universities are increasing the economic evaluation literature related to the region in collaboration with their former colleagues and supervisors. Our findings suggest that the mainstream of academic thinking behind economic evaluations in Africa is likely to respond more to the traditions in America and Europe than those of Sub-Saharan Africa.

The relative strength of South Africa’s research network means that it is well-placed to transfer international evidence and best practices both from Europe and North America, and to other countries in the Sub-Saharan African region. In view of this, we recommend the exploration and application of approaches to stimulate networking among health economic researchers from different African countries, and South Africa should be central to any such efforts. Organizations and institutions from high income countries interested in supporting heath priority-setting in Sub-Saharan Africa could also include promoting collaboration as part of their agendas.

Finally, follow-up studies should aim to capture comprehensively the research networks of all African authors listed in the identified publications, and include an exhaustive systematic review that includes published and grey literature. The significant investment in terms of human resources and time necessary to conduct such a study requires the commitment of international organizations and institutions from developed countries.

## Additional files

Additional file 1: Contains the variables required for generating the visual network maps. (CSV 43 kb) Additional file 2: This is the R code for generating the visual network maps. (R 1 kb) Additional file 3: Database of included studies and characteristics, network articles, authorship and collaborations. (XLSX 182 kb)

## Appendix

**Table 5:** Articles that analysed more than one African country

Countries included in the study	Title of the Article	Year
Botswana/Kenya/South Africa/Tanzania	Giving tranexamic acid to reduce surgical bleeding in sub-Saharan Africa: an economic evaluation	2010
Botswana/Kenya/Tanzania/Uganda/Zambia/Zimbabwe.	Cost-effectiveness of nevirapine to prevent mother-to-child HIV transmission in eight African countries	2004
Kenya/Mozambique/Tanzania/Uganda/Zimbabwe	Health and economic impact of HPV 16/18 vaccination and cervical cancer screening in Eastern Africa	2012
Kenya/Uganda	Economics of switching to second-line antiretroviral therapy with Lopinavir/ritonavir in Africa: estimates based on DART trial results and costs for Uganda and Kenya	2011
Malawi/Mozambique	Predicting trends in HIV-1 sexual transmission in sub-Saharan Africa through the drug resource enhancement against AIDS and malnutrition model: antiretroviral for reduction of population infectivity, incidence and prevalence at the district level.	2012
Malawi/South Africa/Zimbabwe	Cost-effective diagnostic checklists for meningitis in resource-limited settings	2013
Mozambique/Tanzania	Cost-effectiveness of malaria intermittent preventive treatment in infants (IPTi) in Mozambique and the United Republic of Tanzania	2009
Nigeria/Ghana	Cost-effectiveness analysis of unsafe abortion and alternative first-trimester pregnancy termination strategies in Nigeria and Ghana	2010
Nigeria/South Africa	The potential cost and benefits of raltegravir in simplified second-line therapy among HIV infected patients in Nigeria and South Africa	2013
South Africa/Malawi	Expanding ART for treatment and prevention of HIV in South Africa: estimated cost and cost-effectiveness 2011–2050	2012
Swaziland/Lesotho/Botswana/Malawi/Mozambique/Namibia/South Africa/Tanzania/Uganda/Zambia/Zimbabwe/Kenya	Voluntary medical male circumcision: modelling the impact and cost of expanding male circumcision for HIV prevention in eastern and southern Africa	2012
Swaziland/Tanzania/Uganda/Zambia	Assessing effectiveness and cost-effectiveness of concurrency reduction for HIV prevention	2011
Zimbabwe/Uganda	Cost effectiveness analysis of clinically driven versus routine laboratory monitoring of antiretroviral therapy in Uganda and Zimbabwe	2012

**Table 6:** Information extracted from the selected articles

Category	Name of the Variable	Description
GENERALINFORMATION: ARTICLE	Title	Title of the article
	Year (2004–2014)	Publication Year
	Method (cost-effectiveness = 1)	In those cases in which the methodology applied can be classified as cost-effectiveness analysis the value will be equal to 1. If that is not the case, the name of the methodology applied should be included.
	Journal	Journal name
GEOGRAPHIC SCOPE	Country	Countries from the list of the selected countries that are analysed in the document.
	Article focus is on Africa (YES/NO)	Yes = The article analyses only African countries. No = The analysis includes countries inside and outside Africa. In this case only those articles in which at least one of the first three authors is from Africa are included.
DISEASE AND TYPE OF INTERVENTION	Type of Intervention	Based on the classification use by the MRC in its document Outputs, Outcomes and Impact of MRC Research [55]: • Diagnostic Tool – Imaging • Diagnostic Tool - Non-Imaging • Health and Social Care Services • Management of Diseases and Conditions • Preventative Intervention - Behavioural risk modification • Preventative Intervention - Nutrition and Chemoprevention • Preventative Intervention - Physical/Biological risk modification • Products with applications outside of medicine • Support Tool - For Fundamental Research • Support Tool - For Medical Intervention • Therapeutic Intervention - Cellular and gene therapies • Therapeutic Intervention – Complementary • Therapeutic Intervention – Drug • Therapeutic Intervention - Medical Devices • Therapeutic Intervention – Physical • Therapeutic Intervention - Psychological/Behavioural • Therapeutic Intervention – Radiotherapy • Therapeutic Intervention – Surgery • Therapeutic Intervention - Vaccines
	Disease	The analysis is related to the treatment, diagnostic or prevention of the disease that is included in this column.
	Main Conclusion	Use the exact quotation as appeared in the abstract.
AUTHORS’ INFORMATION	Authors	Name of the first three authors.
	Affiliation Country of the First, Second and Third Authors	1) Only the first three authors. 2) In those cases in which the three authors have the same affiliation country, only one country appears in this column.

**Table 7:** Country of Affiliation of the three first authors of the 119 selected articles

At least one African Affiliation*				Affiliation from a Country outside Africa*			
Country	First author	Second author	Third author	Country	First author	Second author	Third author
Congo	0	1	2	Australia	1	1	0
Ethiopia	3	6	2	Belgium	1	0	0
Ghana	2	6	5	Canada	1	0	1
Kenya	5	4	4	Denmark	1	1	1
Lesotho	1	1	0	France	0	1	0
Madagascar	1	1	1	Germany	0	0	1
Malawi	1	3	2	Ireland	2	1	0
Mozambique	0	1	5	Italy	3	2	2
Nigeria	6	9	8	Japan	1	1	1
Seychelles	0	1	0	Myanmar	0	0	1
South Africa	7	8	8	Netherlands	5	3	1
Tanzania	0	0	1	Norway	2	1	1
Uganda	3	7	7	Spain	1	1	0
Zambia	2	2	7	Sweden	0	1	1
Zimbabwe	0	0	2	Switzerland	4	5	5
Botswana/USA	1	0	0	UK	16	12	11
Congo/Belgium	1	0	0	USA	39	31	28
Ethiopia/Norway	1	0	0	France / USA	0	1	1
Ghana/UK	1	1	2				
Mozambique/Spain	1	1	0				
Nigeria/UK	1	0	0				
South Africa/Norway	0	1	0				
South Africa/USA	2	0	1				
Tanzania/Norway	1	2	0				
Tanzania/UK	0	0	1				
Tanzania/UK/Switzerland	1	0	0				
Uganda/UK	1	0	0				
USA/South Africa	0	1	0				
Total	42*	56	58		77	62	55

*Estimated based on the 119 articles. Two papers with the same author were counted twice

**Author selected for the network analysis

Source: Authors’ Elaboration

## Abbreviations

ASERNIP-S: Australian Safety and Efficacy Register of New Interventional Procedures - Surgical
CDR: Centre for Reviews and Dissemination
EQ-5D: EuroQoL 5D
HIV/AIDS: Human immunodeficiency virus/acquired immunodeficiency syndrome
HTA: Health technology assessment
LMIC: Low and middle income countries
MDGs: Millennium Development Goals
MRC: Medical Research Council
NHS EED: National Health Service Economic Evaluation Database
NICE: National Institute for Health and Care Excellence
OHE: Office of Health Economics
QALY: Quality-adjusted life year
SADC: Sub-Saharan African Development Community
